# The long subclinical phase of *Mycobacterium avium* ssp. *paratuberculosis* infections explained without adaptive immunity

**DOI:** 10.1186/s13567-015-0202-3

**Published:** 2015-06-19

**Authors:** Don Klinkenberg, Ad Koets

**Affiliations:** Department of Farm Animal Health, Faculty of Veterinary Medicine, Utrecht University, Yalelaan 7, 3584 CL Utrecht, The Netherlands; Department of Bacteriology and TSE, Central Veterinary Institute of Wageningen UR, Lelystad, The Netherlands; Present address: Centre for Infectious Disease Control, National Institute for Public Health and the Environment, PO Box 1, 3720 AB Bilthoven, The Netherlands

## Abstract

*Mycobacterium avium* ssp. *paratuberculosis* (MAP) is an infection of the ruminant intestine. In cows, a long subclinical phase with no or low intermittent shedding precedes the clinical phase with high shedding. It is generally considered that an adaptive cell-mediated immune response controls the infection during the subclinical phase, followed by unprotective antibodies later in life. Based on recent observations, we challenge the importance of adaptive immunity and instead suggest a role of the structural organization of infected macrophages in localized granulomatous lesions. We investigated this hypothesis by mathematical modelling. Our first model describes infection in a villus, assuming a constant lesion volume. This model shows the existence of two threshold parameters, the MAP reproduction ratio *R*_*MAP*_ determining if a lesion can develop, and the macrophage replacement ratio *R*_*MF*_ determining if recruitment of macrophages is sufficient for unlimited growth. We show that changes in *R*_*MF*_ during a cow’s life – i.e. changes in the innate immune response – can cause intermittent shedding. Our second model describes infection in a granuloma, assuming a growing lesion volume. This model confirms the results of the villus model, and can explain early slow granuloma development: small granulomas grow slower because bacteria leave the granuloma quickly through the relatively large surface area. In conclusion, our models show that the long subclinical period of MAP infection can result from the structural organization of the infection in granulomatous lesions with an important role for innate rather than adaptive immunity. It thus provides a reasonable hypothesis that needs further investigation.

**Electronic supplementary material:**

The online version of this article (doi:10.1186/s13567-015-0202-3) contains supplementary material, which is available to authorized users.

## Introduction

*Mycobacterium avium* ssp. *paratuberculosis* (MAP) causes infection in the ruminant intestine. Cows are most susceptible to become infected in the first half year of their lives [1,2], and infection is characterised by a long sub-clinical phase with no or low intermittent shedding, ending in progressive infection with clinical signs in a small proportion of infected animals [3]. The long sub-clinical phase is generally considered to result from an adaptive T cell mediated cellular immune response [4,5]. This response is thought to keep the infection under control during the sub-clinical phase, without elimination, but fails to do so at a later age. At a later age, typically 3–6 years, antibodies are frequently observed [6], which has led to the hypothesis of a switch from a cellular to a humoral adaptive immune response according to the murine Th1-Th2 paradigm [4,5,7]. An increase in IL-10 production by regulatory T cells or macrophages is considered to cause this switch, by downregulating Th1 responses and stimulating antibody production [8–12]. Immunological data also show that stronger Th1 responses are correlated with increased shedding [6,13,14]. However, recent analyses [15] do confirm that cellular immunity may exert some control over the infection, but this seems to be very different between cows. The clear concept of a protective cellular response followed by a permissive humoral response seems not to hold, at least not for every animal. Similar observations which lead to questioning the relevance of the Th1/Th2 paradigm have been reported for ovine paratuberculosis [14].

A problem of the observations leading to the immune switch hypothesis as described above, is that of causality: histological studies are based on observations on a single point in time, whereas longitudinal observations (immune responses and shedding) are measured on the level of the animal in blood and faeces. The actual infection process of MAP, however, is highly localised with granulomas developing within lymph nodes and the villi of the small intestine [16]. By granuloma in this article we mean simply a localized aggregate of infected and uninfected macrophages, and not per se a traditional granuloma which would be macrophages surrounded by a ring of lymphocytes, and with fibrosis and a central necrotic region. Each granuloma (or lesion) consists of uninfected macrophages and infected macrophages that have structural and intracellular characteristics specific to the granuloma environment. Sometimes these lesions are focal or multi-focal, i.e. small and well-structured with low quantities of MAP, sometimes they are diffuse, i.e. larger with many infected macrophages and higher quantities of MAP [16,17]. In contrast to tuberculosis, relatively few lymphocytes are present in these lesions (compare [6,17] with [18]). When comparing CD4+ T cell counts after immunohistological staining of lesional and non-lesional ileum and jejunum from latent-phase and uninfected control cows, no significant differences are observed. In cows showing clinical signs, the number of CD4+ T cells is even lower compared to the control and latent-phase cows [6]. In a study in which CD4+ T cells were depleted in infected calves using monoclonal antibodies, no effect on the course of the disease was observed despite documented T cell depletion [19]. Finally, T cell immunosuppressive treatment of latent-phase MAP-infected cows does not accelerate disease progression due to MAP, where it does result in BHV1 reactivation and an increase in shedding of worm eggs and oocysts [20]. Of course, adaptive immune responses – predominantly developed in the gut draining lymph nodes – may well be able to deal with infected macrophages as such, but apparently this does not lead to a clear correlation with shedding. This lack of control of shedding may result from a lack of local accumulation in intestinal tissue or from MAP actively counteracting T cell attraction [21] and antigen presentation [22,23].

This leads to the following research question: can the dynamics of the localised granulomatous lesion itself explain the typical long latent period of a MAP infection, independent of the associated adaptive immune response? The spatial characteristics of the localised structure may limit the opportunity of MAP to infect new macrophages upon release from infected cells, especially if there is no inflammatory reaction attracting monocytes to the lesion as MAP intracellular infection prevents generation of inflammatory signals by infected macrophages [21]. Of course, it is necessary that dynamics of individual lesions is to some extent synchronized throughout the intestine to explain excretion dynamics, but that is not unlikely: first, many local infections start at about the same age, within a time window of several months during the first half year of life; and second, even if the infection process occurs localized, parameters governing this process may well be controlled on the level of the animal as a whole, e.g. hormonal changes during pregnancy and calving [24–26].

A useful method to study questions as the above, by identifying the consequences of a hypothesis to make testable predictions, is mathematical modelling [27]. Although there are many modelling studies of MAP epidemiology and transmission (e.g. [28,29]), there is only one within-host model, which focused on the role of adaptive immunity [30]. Most models of within-host *tuberculosis* (TB) [31–34] were also used to study the role of adaptive immunity, which is exactly what we want to leave out for our question. Another problem of these models is their detail in including various immune cells and cytokines, which was possible because of the availability of much quantitative TB data, whereas MAP data are much scarcer. A notable exception is a model of Gammack et al. [35], who used a relatively simple spatial granuloma model to predict conditions for controlled and uncontrolled granuloma growth. However, that model did not address the long subclinical phase of infection.

In this paper we construct two models to explore the possibility of a long subclinical phase for MAP infection as a result of the spatial characteristics of localised granulomatous infection, in the absence of a local adaptive immune response. Both models are based on the same simple description of the local infection process, but each with a different assumption on how the infection occupies space within a villus. First, in the *villus model* it is assumed that the infection is present in the whole villus and that the local density of macrophages and bacteria changes during the course of the infection, but not the size of the lesion. Second, in the *granuloma model* it is assumed that the macrophages clump together, forming a granuloma, and that the number of macrophages and bacteria changes during the course of the infection, but not the local density of macrophages inside the lesion. With both models, we identify conditions under which a long subclinical phase could arise independently of an adaptive immune response acting on the lesion, which we then address in the discussion.

## Materials and methods

### The model for MAP dynamics in a villus

**Figure 1:**
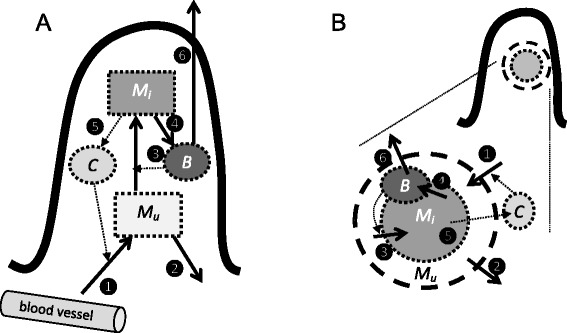
**Sketch of the villus and granuloma models.** In both models, *M*_*u*_ is the number of uninfected macrophages, *M*_*i*_ is the number of infected macrophages, *B* is the number of free bacteria, and *C* is the amount of cytokines. The processes in both models are (1) inflow of uninfected macrophages, both normal “background” inflow and additional recruitment proportional to the amount of cytokines; (2) outflow of uninfected macrophages; (3) infection of uninfected macrophages by free bacteria; (4) bursting of infected macrophages and release of free bacteria; (5) outflow of cytokines produced by infected macrophages; (6) outflow of free bacteria resulting in phagocytosis and excretion. All rates are density-dependent. In the villus model (**A**) the volume remains constant and the densities are proportional to the numbers of cells and amount of cytokines. In the granuloma model (**B**) the density of macrophages remains constant and the volume is determined by the number of macrophages; the densities are equal to the numbers of cells and amount of cytokines divided by the granuloma volume; some rates are proportional to the surface area (1, 2, 5, 6).

We first describe the model for local MAP dynamics in a single villus (*villus model*), which is adjusted to describe the dynamics in a granuloma (*granuloma model*) in the next section (Figure 1). In the villus model, the volume is kept constant so that the numbers of cells in the lesion are proportional to their concentrations. The full villus model (Figure 1A) describes the dynamics of three populations of cells: uninfected macrophages *M*_*u*_, infected macrophages *M*_*i*_ and free bacteria *B*, all expressed in numbers per lesion. In addition, the model keeps track of the amount of monocyte-attracting cytokines *C*, such as IL-1 and chemokine CCL2, in arbitrary units in the lesion. T cell related cytokines were not included into the model because of our focus on the role of macrophages, but their action could be interpreted through individual model parameters, e.g. the bacterial killing rate:

1 $$ \begin{array}{l}{M}_u\hbox{'}=i-d{M}_u+aC-cB{M}_u\\ {}{M}_i\hbox{'}=cB{M}_u-b{M}_i\\ {}B\hbox{'}=bQ{M}_i-eB\\ {}C\hbox{'}=r{M}_i-mC\end{array} $$

Because the volume is kept constant, all density-dependent processes occur at rates proportional to the numbers of cells (or amount of cytokines). Independently of infection, uninfected macrophages enter the site at rate *i* (number of cells per day) and leave at rate *d* (per cell per day). The presence of cytokines attracts new monocytes to the site at rate *a* per unit *C* per day, which become additional uninfected macrophages. Uninfected macrophages are infected by free bacteria, at rate *c* per bacterium per day. Infected macrophages do not leave the site; MAP multiplies inside the macrophages until they burst and release the bacteria. Bursting occurs at rate *b* per day. Bursting results in the release of *Q* free bacteria. These free bacteria decrease in number by infecting new macrophages, by being killed by uninfected macrophages, and by release into the gut. We assume that reduction of free bacteria by infection of new macrophages is negligible compared to killing and release, that all processes together occur at rate *e* per day. Finally, cytokines *C* are produced by infected macrophages at rate *r* and leave the site by diffusion at rate *m*.

In order to reduce the model dimension, we take a quasi-steady state assumption because the rates at which the free bacteria and cytokines are removed from the villus (*e* and *m*, order of hours) are much higher than the turnover rates of macrophages (*d* and *b*, order of weeks [R49-51]). Thus, by assuming that *B*’ = 0 and *C*’ = 0, we can work with only the equations for *M*_*u*_ and *M*_*i*_, in which *B* = *bQM*_*i*_/*e* and *C* = *rM*_*i*_/*m*:

$$ \begin{array}{l}{M}_u\hbox{'}=i-d{M}_u+ar{M}_i/m-cbQ{M}_i{M}_u/e\\ {}{M}_i\hbox{'}=cbQ{M}_i{M}_u/e-b{M}_i\end{array} $$

We now define the following quantities:

- *M*_*u*_^0^ = *i*/*d* is the steady state number of uninfected macrophages in an infection free villus.
- *R*_*MF*_ = *ar*/*bm* is the macrophage replacement ratio. Each infected macrophage produces *r/b* units of cytokine during their lifetime, which remains in the villus for 1/*m* days on average, during which it attracts *a* uninfected macrophages per day. That means that each infected macrophage results in the attraction of *R*_*MF*_ new macrophages to the villus.
- *R*_*MAP*_ = (*cQ*/*e*) × *M*_*u*_^0^ is the MAP basic reproduction ratio. Each infected macrophage releases *Q* free bacteria, which remain in the villus for 1/*e* days on average, during which they infect *cM*_*u*_ uninfected macrophages. *R*_*MAP*_ is defined for the onset of the infection, so with *M*_*u*_ = *M*_*u*_^0^, it can be interpreted as the number of newly infected macrophages resulting from one infected macrophage.

Thus we arrive at the reduced and final villus model:

2 $$ \begin{array}{l}{M}_u\hbox{'}=d\left({M}_u^0-{M}_u\right)+b{R}_{MF}{M}_i-b{\scriptscriptstyle \frac{R_{MAP}}{M_u^0}}{M}_i{M}_u\\ {}{M}_i\hbox{'}=b{\scriptscriptstyle \frac{R_{MAP}}{M_u^0}}{M}_i{M}_u-b{M}_i\end{array} $$

Aside from *R*_*MF*_ and *R*_*MAP*_, the role of which will be addressed in the model analysis, the model contains three parameters:

- *d* = 0.04, the turnover rate of uninfected macrophages. This value reflects an average residency of 25 days in the villus, after which the macrophage dies or goes back into circulation. This corresponds to macrophage turnover rates measured in various tissues in mice [36–39].
- *M*_*u*_^0^ = 10, the number of macrophages in an infection free villus. This value is a conservative estimate for the mouse intestine [39].
- *b* = 0.04, the bursting rate of infected macrophages. For lack of data, we assume this to be equal to *d*, which seems reasonable: it would match ten rounds of bacterial doubling each taking 2–3 days, resulting in 1000 bacteria per cell. This is in line with histological observations (Koets, unpublished data).

### The model for MAP dynamics in a granuloma

For adaptation of the villus model to a model for a single granuloma, we assume that the density of cells is constant and that the volume of the lesion changes; thus, we take the dimensions of the granuloma into account. For simplicity, we assume a perfect sphere with the volume determined by the total number of uninfected and infected macrophages, *V* = *ρ*(*M*_*u*_ + *M*_*i*_), with *ρ* being the unit volume taken per macrophage inside a granuloma. The surface area can thus be calculated as *A* = (36*π*)^1/3^*V*^2/3^ = *kV*^2/3^, with *k* = (36*π*)^2/3^. Now we take two steps: first, the rates of processes that depend on densities rather than numbers of macrophages and bacteria (infection of uninfected macrophages, and inflow and outflow of macrophages, bacteria, and cytokines), are adjusted by division by *V*. Second, the rates of processes that happen across the surface area of the granuloma (inflow and outflow of macrophages, bacteria, and cytokines) are made proportional to this surface area. Thereby, the dimensions of the parameters *i*, *d*, *a*, *e*, and *m* change to [per day, per unit surface area]. Thus, the granuloma model reads:

3 $$ \begin{array}{l}{M}_u\hbox{'}=iA-d\frac{M_u}{V}A+a\frac{C}{V}A-c\frac{B}{V}{M}_u\\ {}{M}_i\hbox{'}=c\frac{B}{V}{M}_u-b{M}_i\\ {}B\hbox{'}=bQ{M}_i-e\frac{B}{V}A\\ {}C\hbox{'}=r{M}_i-m\frac{C}{V}A\end{array} $$

Next, we make the same quasi-steady state assumption as in the villus model. With the concentration of bacteria equal to *B*/*V* = *bQM*_*i*_/(*eA*) and the concentration of cytokines equal to *C*/*V* = *rM*_*i*_/(*mA*). Then, we define the following quantities:

- *R*_*MF*_ = *ar*/*bm*, is the macrophage replacement ratio as in the villus model.
- *T*_*MAP*_ = *cQ*/*e*, is the MAP transmission efficiency. Similar to *R*_*MAP*_ in the villus model, *T*_*MAP*_ can be interpreted as the number of newly infected macrophages resulting from one infected macrophage in the absence of any (other) infected macrophages, but this situation never occurs as a granuloma does not exist in the absence of infected macrophages. Thus, *T*_*MAP*_ should be more loosely interpreted as a measure of MAP transmissibility between macrophages within the granuloma.

Thus, we arrive at the reduced and final granuloma model:

4 $$ \begin{array}{l}{M}_u\hbox{'}=iA-dA\frac{M_u}{V}+b{R}_{MF}{M}_i-b\frac{T_{MAP}}{A}{M}_i{M}_u\\ {}{M}_i\hbox{'}=b\frac{T_{MAP}}{A}{M}_i{M}_u-b{M}_i\end{array} $$

This model contains four parameters aside from *T*_*MAP*_ and *R*_*MF*_:

- *ρ* = 1, the volume of the granuloma per macrophage present, in arbitrary units.
- *d* = 0.04, as in the villus model.
- *i*, the rate by which uninfected macrophages enter the granuloma by background macrophage movement. It should be smaller than *d*/*ρ*, otherwise granulomas of uninfected macrophages would spontaneously arise. As a default in simulations, we will use *i* = 0.038 (arbitrary choice).
- *b* = 0.04, as in the villus model.

### Model analysis

The aim of the model analyses is to determine for both models what types of behaviour they can display in terms of bacterial replication and resulting bacterial load in the lesion. We seek to answer these questions: will MAP disappear, will it grow until a stable steady state, or will it grow indefinitely so that it outgrows the villus and disrupts the structure of the intestinal wall? The conditions for these types of behaviour will be determined by mathematical analysis and numerical simulations with the above parameter values. All numerical simulations were done in Mathematica 7.0 [40].

## Results

### The villus model

Mathematical analysis (see Additional file 1) of the villus model (Equation 2) shows that two steady states are possible: the infection-free steady state with *M*_u_^*^ = *M*_*u*_^0^ and *M*_i_^*^ = 0; and an infected steady state with

5 $$ \begin{array}{l}{M}_u^{*}=\frac{M_u^0}{R_{MAP}}\\ {}{M}_i^{*}=\frac{M_u^0}{R_{MAP}}\frac{d\left({R}_{MAP}-1\right)}{b\left(1-{R}_{MF}\right)}\end{array} $$

From Equation 5 it is easily seen that the infected steady state is positive – thus exists only – if either *R*_*MAP*_ *>* 1 and *R*_*MF*_ *<* 1, or if *R*_*MAP*_ *<* 1 and *R*_*MF*_ *>* 1. Stability analysis of these steady states (see Additional file 1) shows that the infection-free steady state is locally stable if *R*_*MAP*_ *<* 1 (less than one newly infected macrophage resulting from one infected macrophage). The infected steady state is stable only if *R*_*MAP*_ *>* 1 and *R*_*MF*_ *<* 1 (each infected macrophage attracts less than one monocyte to the villus through its produced cytokines).

**Figure 2:**
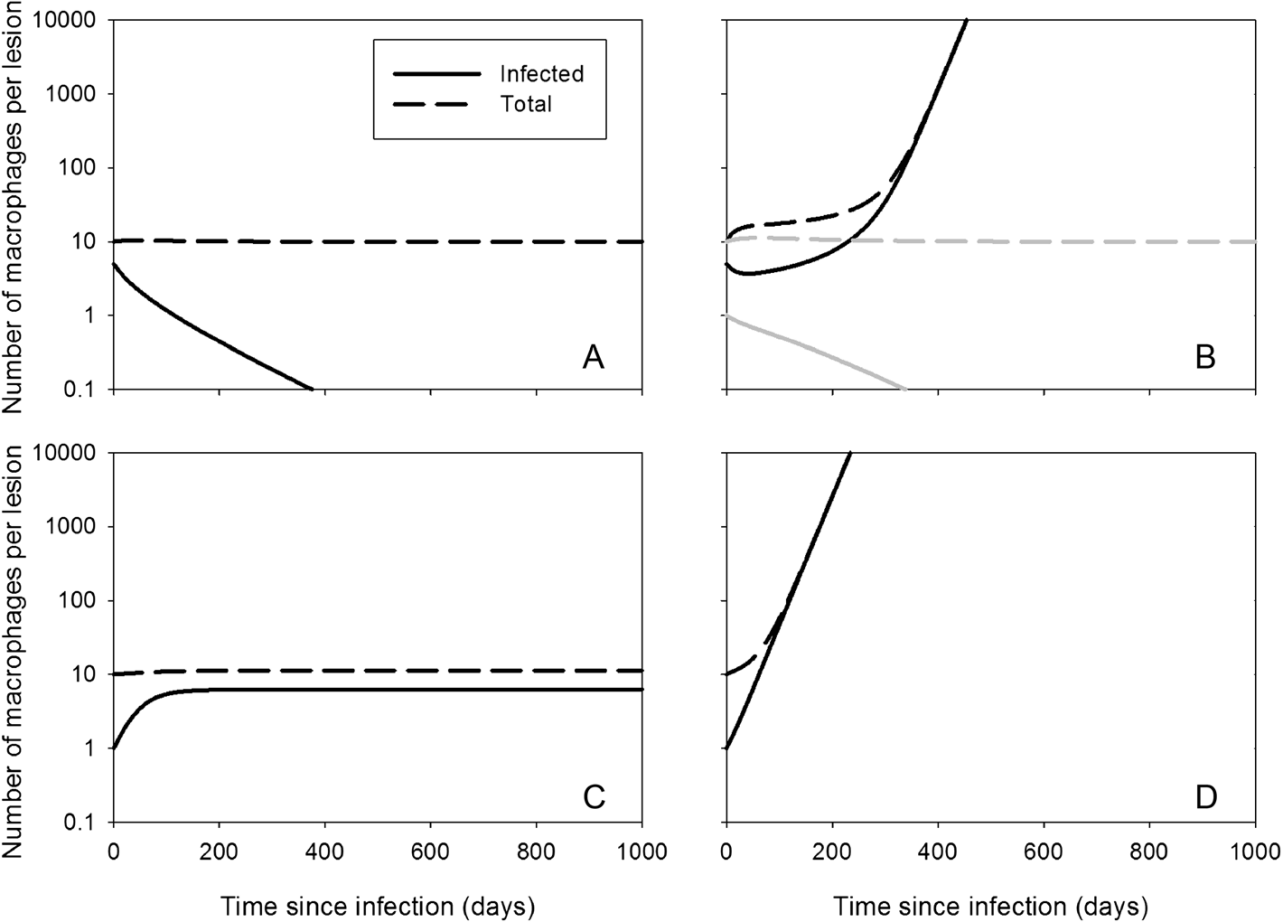
**MAP dynamics with the villus model.** Simulations with the villus model (Equation 2), with parameter values *d* = 0.04, *M*_*u*_^0^ = 10, and *b* = 0.04. Other parameters: (**A**) *R*_*MAP*_ = 0.8, *R*_*MF*_ = 0.2; (**B**) *R*_*MAP*_ = 0.8, *R*_*MF*_ = 2; (**C**) *R*_*MAP*_ = 0.8, *R*_*MF*_ = 0.2; (**D**) *R*_*MAP*_ = 0.8, *R*_*MF*_ = 0.2. Initial conditions: (*M*_*u*_, *M*_*i*_) = (5, 5) in (**A**) and (**B**, black curve); (*M*_*u*_, *M*_*i*_) = (9, 1) in (**B**, grey curve), (**C**), (**D**).

These existence and stability criteria result in four different situations, illustrated in Figure 2. First, if both *R*_*MAP*_ *<* 1 and *R*_*MF*_ *<* 1, no infected steady state exists and MAP will disappear irrespective of the initial condition (Figure 2A). Second, if *R*_*MAP*_ *<* 1 and *R*_*MF*_ *>* 1, an infected steady state does exist but it is not stable. This means that the long-term outcome depends on the initial condition: if there are few infected macrophages, MAP will disappear, but if there are many infected macrophages MAP will grow indefinitely (Figure 2B). In biological terms it means that the lesion outgrows the villus, which makes the model invalid for further description of the lesion. The threshold number (between “few” and “many”) is determined by the exact parameter values. Third, if *R*_*MAP*_ *>* 1 and *R*_*MF*_ *<* 1, the infection-free steady state is not stable and the infection will go to a stable infected steady state for all initial conditions (Figure 2C). Fourth, if *R*_*MAP*_ *>* 1 and *R*_*MF*_ *>* 1, the infection will grow indefinitely and outgrow the villus with any initial condition as long as *M*_*i*_ > 0 (Figure 2D).

**Figure 3:**
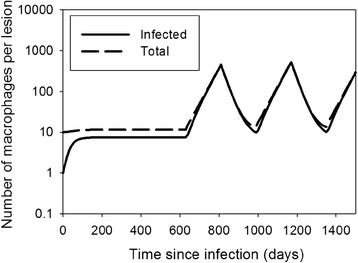
**MAP intermittent shedding dynamics with the villus model.** Simulations with the villus model (Equation 2), with parameter values *d* = 0.04, *M*_*u*_^0^ = 10, *b* = 0.04, *R*_*MAP*_ = 2.5. The parameter *R*_*MF*_ = 0.2 before day 630, between days 810 and 990, and between days 1170–1350, otherwise *R*_*MF*_ = 1.5. This reflects a cow with yearly calving and higher *R*_*MF*_ three months before and three months after calving.

The numerical simulations show that if the infection grows indefinitely (if *R*_*MF*_ *>* 1), which would reflect the development of pathology and clinical signs, it does so immediately if *R*_*MAP*_ *>* 1 (Figure 2D), but with a delay if *R*_*MAP*_ *<* 1 (Figure 2B). This delay could be interpreted as a subclinical phase in which efficient recruitment (*R*_*MF*_ *>* 1) just outbalances inefficient infection (*R*_*MAP*_ *<* 1). However, this is biologically not a very likely scenario as it would result in pathology and clinical signs relatively quickly in all cows, whereas in reality most remain subclinical for many years. However, as long as *R*_*MF*_ *<* 1, the number of infected macrophages in the villus remains small and the infection is kept limited. It could be that this is the situation during the subclinical phase of the infection: MAP is present in the intestine but only locally in small quantities. If conditions change which causes *R*_*MF*_ to increase across many villi, e.g. when leucocyte levels are higher, when cows are more susceptible to infections in the last trimester of pregnancy and the first three months of lactation [41], that could stimulate recruitment of macrophages and thereby multiplication of MAP. If this only happens briefly it could result in a short period of higher shedding, but if it happens for a longer period of time it could end the subclinical phase and exponential replication of bacteria could result in high shedding and pathology, as observed in cows progressing to the clinical stage of disease. Figure 3 shows a simulation of a possible course of the infection under this scenario, with parturitions every year from the second year onwards.

### The granuloma model

Mathematical analysis of the granuloma model shows the possible existence of three steady states: the infection-free steady state with both *M*_u_^*^ = 0 and *M*_i_^*^ = 0; and two infected steady states. These infected steady states will here be referred to by the volume of the granulomas, *V*_small_^*^ and *V*_large_^*^ (equal to *ρ*(*M*_u_^*^ + *M*_i_^*^)). In both steady states, the number of uninfected macrophages is proportional to the surface area of the granuloma: *M*_u_^*^ = A^*^/*T*_*MAP*_. Although the model is not explicitly spatial (it does not describe where the infected and uninfected macrophages are), this relation suggests that the uninfected macrophages are situated at the surface of the granuloma, and that the thickness of this layer of cells is determined by *T*_*MAP*_, which is the efficiency by which they are infected.

Existence and stability analysis of all steady states (see Additional file 1) shows that the infection-free steady state always exists, and that it is always locally stable. The infected steady state *V*_small_^*^ exists if conditions “are favourable” (for exact conditions see Additional file 1), which means that *T*_*MAP*_ is high enough in relation to the inflow and outflow of uninfected macrophages (*i*, *R*_*MF*_, *d*), and in relation to the bursting rate *b*. This steady state *V*_small_^*^ is always unstable. The infected steady state *V*_large_^*^ exists only if *V*_small_^*^ exists and if *R*_*MF*_ < 1; this steady state is always stable.

**Figure 4:**
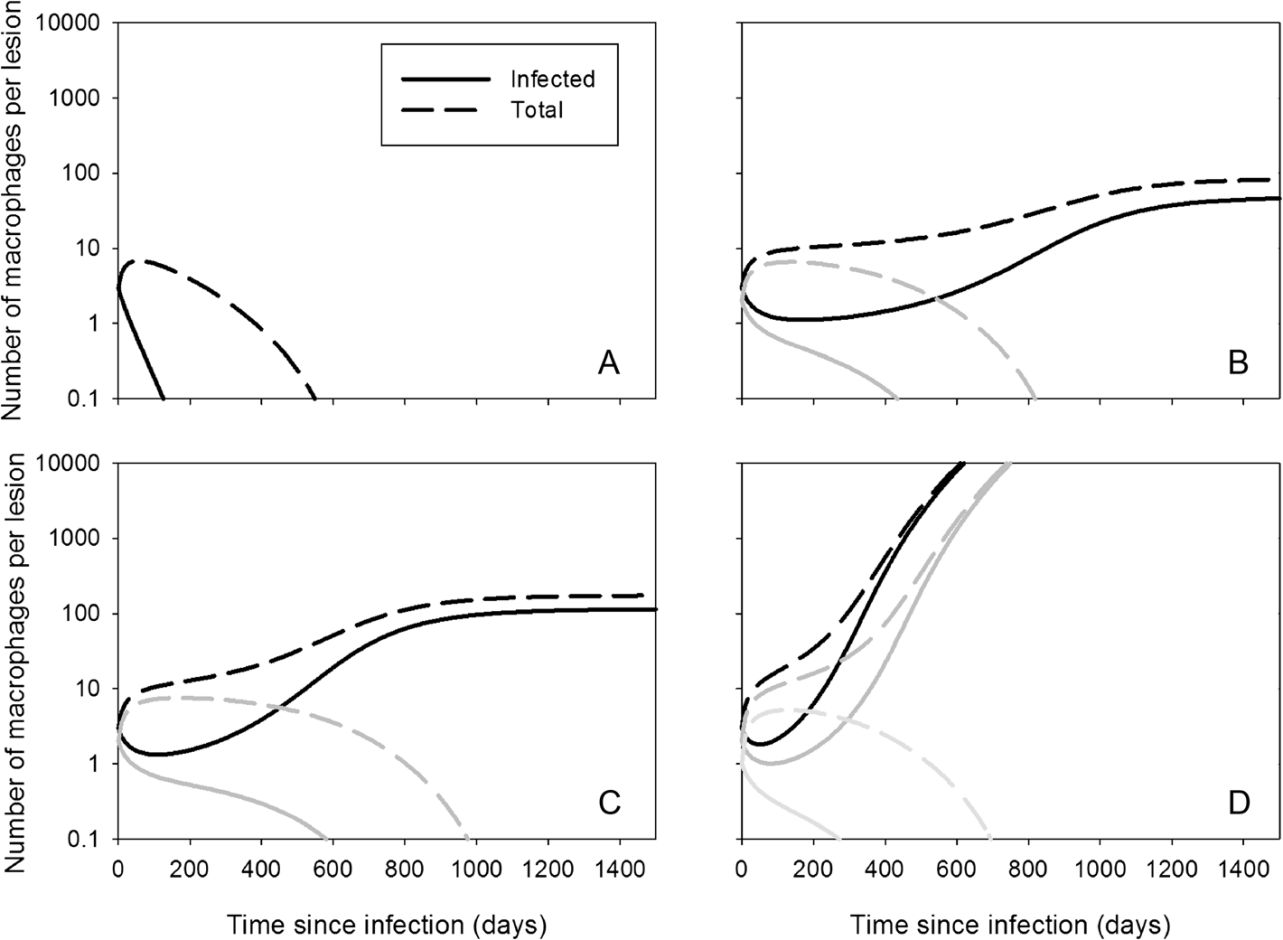
**MAP dynamics with the granuloma model.** Simulations with the granuloma model (Equation 4), with parameter values *d* = 0.04, *i* = 0.38, *ρ* = 1, and *b* = 0.04. Other parameters: (**A**) *T*_*MAP*_ = 1.0, *R*_*MF*_ = 0; (**B**) *T*_*MAP*_ = 2.5, *R*_*MF*_ = 0; (**C**) *T*_*MAP*_ = 2.5, *R*_*MF*_ = 0.2; (**D**) *T*_*MAP*_ = 2.5, *R*_*MF*_ = 1.1. Initial conditions: (*M*_*u*_, *M*_*i*_) = (0, 3) (black curves); (*M*_*u*_, *M*_*i*_) = (0, 2) (dark grey curves) (*M*_*u*_, *M*_*i*_) = (0, 1) (light grey curve).

These existence and stability criteria result in three different situations, illustrated in Figure 4. First, if conditions are unfavourable for MAP (*T*_*MAP*_ too low), the only existing and stable steady state is the infection-free steady state only (Figure 4A). Second, if conditions are more favourable for MAP, and *R*_*MF*_ < 1, both steady states *V*_small_^*^ and *V*_large_^*^ exist. Then, the steady state *V*_small_^*^ is unstable and acts to divide the fate of developing granulomas: if a starting granuloma has more infected and uninfected macrophages than in this steady state, the granuloma will grow; otherwise it will disintegrate. Figures 4B and 4C show the dynamics of disintegrating and growing granulomas (determined by the initial condition) for two different values of *R*_*MF*_ (0 and 0.2). The plots show that growing granulomas linger for a long time around steady state *V*_small_^*^ (about 10 macrophages, of which 1 infected), before they grow more quickly towards *V*_large_^*^. Third, if *R*_*MF*_ > 1, there is no steady state *V*_large_^*^ and growing granulomas will grow indefinitely and outgrow the villus (Figure 4D).

**Figure 5:**
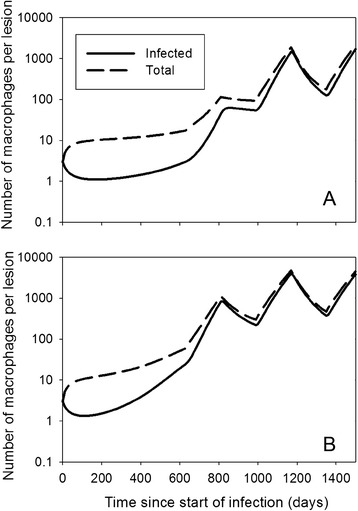
**MAP intermittent shedding dynamics with the granuloma model.** Simulations with the granuloma model (Equation 4), with parameter values *d* = 0.04, *i* = 0.38, *ρ* = 1, and *b* = 0.04. (**A**) The parameter *R*_*MF*_ = 0 before day 630, between days 810 and 990, and between days 1170–1350, otherwise *R*_*MF*_ = 1.1. (**B**) The parameter *R*_*MF*_ = 0.2 before day 630, between days 810 and 990, and between days 1170–1350, otherwise *R*_*MF*_ = 1.1. This reflects a cow with yearly calving and higher *R*_*MF*_ three months before and three months after calving.

The numerical simulations show that if the granuloma does not disintegrate, it does not always grow quickly either: the volume can remain around *V*_small_^*^ for a long time, e.g. 2 years in Figure 4B, depending on parameter values and initial conditions. This may explain part of the subclinical phase, but on top of that – as in the villus model – it may also be that *R*_*MF*_ varies during the life of a cow. Figure 5 shows simulations of possible courses of the infection under the scenario that *R*_*MF*_ is higher for about 6 months each year around parturition, with all other parameter values as in Figures 4B and C.

## Discussion

Infection of cows with MAP is characterised by a long subclinical period with no, low, or intermittent shedding, followed in some individuals by progressive development of the infection with higher shedding levels. In this paper we explored the possibility that this long subclinical phase is not explained by an adaptive immune response, but by the characteristic structural organisation of granulomatous lesions in the intestinal wall. For this aim, we built two mathematical models for a single lesion. Both models describe the infection mechanism with the same cell populations – uninfected macrophages, infected macrophages, and free MAP – but different assumptions about local densities of infected cells and the volume of the lesion. In the villus model, the volume was fixed (a single villus) so that cell densities could change. In the granuloma model the density of macrophages was fixed, and the volume of the granuloma proportional to the number of macrophages. In the granuloma model, inflow and outflow of macrophages, bacteria, and cytokines occurred at rates proportional to the surface area of the granuloma, for which a perfect sphere was assumed.

Both models predict that a threshold parameter *R*_*MAP*_ or *T*_*MAP*_ will be important in early infection, as it determines whether an animal can become infected upon ingestion of MAP. The two models are different in predicting the fate of an early lesion when it starts with very few (e.g. one) infected macrophages. With *R*_*MAP*_ (villus model) clearly defined as the number of newly infected macrophages produced by one infected macrophage, a lesion will always develop if *R*_*MAP*_ > 1. *T*_*MAP*_ (granuloma model) is less clearly defined and small granulomas are predicted always to disintegrate. However, *T*_*MAP*_ does determine the possibility of granuloma formation if it starts with some clustered macrophages right from the start, if bacteria would enter a villus and infect a couple of macrophages that are in close vicinity.

Both *R*_*MAP*_ and *T*_*MAP*_ show that initial infection control depends on a balance between availability of macrophages, rate of macrophage infection by bacteria, number of bacteria released from an infected macrophage, and bacterial clearance rate. This is similar to the results of Gammack et al. [35], who had a very different model formulation for TB granulomas, but found that a balance of phagocytosis, intracellular killing and intracellular bacterial growth determined infection control. Later more detailed TB models included dynamics of classically activated macrophages (phagocytosing and killing) and alternatively activated macrophages (susceptible for TB infection), showing control of TB by cytokines steering macrophage development towards classic activation [32,33]. In our model this distinction is not made, but the fact that *R*_*MAP*_ and *T*_*MAP*_ depend on availability of macrophages for infection and bacterial clearance shows the importance of the actual role of macrophages. Also for MAP, it has been shown that macrophages can be more permissive or become active in phagocytosis [8,42]. However, a model explicitly describing these macrophage populations, and how activation takes place (e.g. by Th1 cytokines), requires more (quantitative) knowledge on this activation process.

Both models also predict the existence of a second threshold parameter, the recruitment ratio of macrophages *R*_*MF*_, defined as the number of uninfected macrophages attracted to the lesion by one infected macrophage, through its excreted cytokines. If *R*_*MF*_ < 1 lesions will dissolve or go to a stable steady state, depending on *R*_*MAP*_ or *T*_*MAP*_. If *R*_*MF*_ > 1, the number of infected macrophages can grow indefinitely, even if *R*_*MAP*_ < 1, but then there should be sufficient infected macrophages to start with. *R*_*MF*_ combines several infection processes that appear important in keeping granulomas restrained: production and outflow of cytokines, rate of attraction of macrophages by cytokines, and bursting rate of infected macrophages. Gammack et al. [35] did not find this second threshold in their model, possibly because they implicitly assumed unlimited availability of macrophages, which led them to conclude that adaptive immunity is necessary for containment. Our results suggest a possibly important role of the host’s innate immune responsiveness through cytokine production and macrophage cytokine sensitivity, that could provoke an inflammatory response at the lesion site. It could be envisaged that *R*_*MF*_ varies throughout a cow’s life, e.g. in lactating cows due to endocrinological changes during pregnancy and lactation [24,25], and that this variation could explain periods of intermittent shedding, followed by silent phases, and possibly even the end of the latent phase if *R*_*MF*_ remains elevated (Figures 3 and 5). It would mean that it is innate rather than adaptive immune that is responsible for the long subclinical phase with intermittent shedding.

A possible explanation for the early part of the subclinical phase, when shedding levels are so low that infection is hardly ever detected, is provided by the granuloma model. It concerns the time before the stable low shedding phase is reached, even before the possible intermittent shedding as explained above. Mechanistically, this very early dynamics can be explained by considering the three-dimensional structure of the granuloma. As the steady-state solutions indicate, the number of uninfected macrophages is proportional to the surface area of the granuloma, which means that in a small granuloma most macrophages are uninfected. They remain uninfected because the concentration of bacteria is low: there are few infected macrophages and free bacteria quickly leave the granuloma through the surface area which is large relative to the volume. This results in a long phase with an almost stable granuloma size. However, the granuloma does grow slowly and at some point, infection occurs sufficiently efficient to infect most macrophages and start rapid growth of the granuloma (Figures 4 and 5). When this early subclinical phase is followed by the regular changes in *R*_*MF*_ due to hormonal changes during pregnancy and around calving, shedding could follow patterns as in Figure 5: first very low due to the granuloma structure, then alternating between intermediate and high due to changes in *R*_*MF*_. Interestingly, this size effect in models of spheres resulting from changes in efficiency of diffusion is well known from models for early tumour growth, where the result is opposite: tumours first grow quickly but come to a halt because of lack of nutrients that have to diffuse from outside [43].

To extrapolate dynamics in a single lesion to excretion patterns, we assume that the dynamics in many lesions is synchronized to some extent, for which we used two arguments: synchronization because parameter values change due to systemic changes in the animal [24–26] and calves are infected within a relatively small time window [1,2]. A third, observational argument is that post-mortem observations on infected animals show little variation in type of lesions throughout the intestine within a single cow, but much variation between cows [17]. However, synchronization does not mean that all lesions are identical [44,45]. Recent non-human primate TB data show that lesions start with one bacterium after low-dose inoculation and reach similar maximum burdens, but vary substantially within the host in their rate of progression [45]. This may suggest that local factors play an important role, but stochastic effects due to low numbers or re-infection later in life could also play a role. Further investigation is needed to understand this heterogeneity.

One aspect of observed excretion patterns is not explained by our models: the initial low-level shedding in the first couple of months after infection [15,46,47]. This was not to be expected, because our models describe dynamics of a single lesion and in this early phase lesions are still being formed. Possibly, in young calves many macrophages are infected as a result of the many Peyer’s patches in the ileum, causing early multiplication and shedding but not all growing into lesions. That could be explained by the granuloma model which predicts that a critical number of infected macrophages needed for lesions to grow. High inoculation doses in these experiments could have played a role as well.

In conclusion, our models show that the long subclinical phase during MAP infection can result from the structural organisation of the infection in granulomatous lesions, without an important role for adaptive immunity. The early subclinical phase can be explained by the three-dimensional organisation in granulomas: small granulomas grow slower because their surface area is relatively large, which causes free bacteria to leave quickly. The later phase of intermittent shedding can be explained by changes in the efficiency of macrophage recruitment to the lesions, thus by changes in the innate immune response rather than the adaptive response. This variation in immunocompetence during a cow’s life could for instance be related to hormonal changes during pregnancy or lactation. Although our model cannot prove that adaptive immunity does not play any role, it does to provide a reasonable alternative which suggests that investigation of local factors and innate immunity at the lesion site may provide a better understanding of paratuberculosis pathogenesis.

## Additional file

Additional file 1:
**Analysis of the mathematical models.** Details on the steady state analysis of the mathematical models: conditions for existence and stability.
