# Supplementary material for: The long subclinical phase of Mycobacterium avium ssp. paratuberculosis infections explained without adaptive immunity
Source: Vet Res. 2015 Jun 19;46(1):63. doi: 10.1186/s13567-015-0202-3 (PMC4473850; doi:10.1186/s13567-015-0202-3)
Supplement: Additional file 1: — Analysis of the mathematical models. Details on the steady state analysis of the mathematical models: conditions for existence and stability. [file 13567_2015_202_MOESM1_ESM.docx]

**Additional file 1**

The villus model

The reduced and final model for infection in a single villus (Eq. (2) in the main text) is

Steady states

By solving $M_{u}'=0$ and $M_{i}'=0$, we find the steady states of the villus model:

- **steady state V1**: $\left(M_{u}^{*},M_{i}^{*}\right)_{\text{V1}}=\left(M_{u}^{0},0\right)$
- **steady state V2**: $\left(M_{u}^{*},M_{i}^{*}\right)_{\text{V2}}=\left(\frac{M_{u}^{0}}{R_{MAP}},\frac{dM_{u}^{0}\left(1-1/R_{MAP}\right)}{b\left(1-R_{MF}\right)}\right)$

Steady state V1 is the infection-free steady state, and steady state V2 is an infected steady state. Because steady state numbers of cells cannot be negative, the infected steady state V2 only exists if either $R_{MAP}>1$ and $R_{MF}<1$, or if $R_{MAP}<1$ and $R_{MF}>1$.

Local stability of the steady states

Local stability analysis is done by determining the Jacobian matrices in the two steady states, and calculating the traces and determinants of these matrices. Steady states are stable if the trace is negative and the determinant is positive.

For steady state V1, the Jacobian matrix is

$$J_{\text{V1}}=\left(\begin{matrix} -d & b\left(R_{MF}-R_{MAP}\right) \\ 0 & b\left(R_{MAP}-1\right) \end{matrix}\right)$$

so the trace and determinant are

$$\begin{matrix} \text{Tr}_{\text{V1}}=-d+b\left(R_{MAP}-1\right) \\ \text{Det}_{\text{V1}}=-bd\left(R_{MAP}-1\right) \end{matrix}$$

For $\text{Det}_{\text{V1}}$ to be positive, $R_{MAP}<1$, in which case $\text{Tr}_{\text{V1}}$ is negative.

**Conclusion about steady state V1**: the infection-free steady state V1 always exists, and is stable if $R_{MAP}<1$.

For steady state V2, the Jacobian matrix is

$$J_{\text{V2}}=\left(\begin{matrix} -d-d\left(\frac{R_{MAP}-1}{1-R_{MF}}\right) & b\left(R_{MF}-1\right) \\ d\left(\frac{R_{MAP}-1}{1-R_{MF}}\right) & 0 \end{matrix}\right)$$

so the trace and determinant are

$$\begin{matrix} \text{Tr}_{\text{V2}}=-d-d\left(\frac{R_{MAP}-1}{1-R_{MF}}\right) \\ \text{Det}_{\text{V2}}=bd\left(R_{MAP}-1\right) \end{matrix}$$

For the determinant to be positive, $R_{MAP}>1$. If $R_{MAP}>1$, then $R_{MF}<1$ for the steady state to exist at all (to be positive), in which case $\text{Tr}_{\text{V2}}<0$.

**Conclusion about steady state V2**: the infected steady state V2 exists and is stable if $R_{MAP}>1$ and $R_{MF}<1$. The infected steady state exists and is unstable if $R_{MAP}<1$ and $R_{MF}>1$. In all other cases steady state V2 does not exist

The granuloma model

The reduced and final model for infection in a single granuloma (Eq. (4) in the main text) is

$$\begin{matrix} M_{u}'=iA-dA\frac{M_{u}}{V}+bR_{MF}M_{i}-b\frac{T_{MAP}}{A}M_{i}M_{u} \\ M_{i}'=b\frac{T_{MAP}}{A}M_{i}M_{u}-bM_{i} \end{matrix}$$

In this model, $V=\rho\left(M_{u}+M_{i}\right)$ and $A=kV^{2/3}$, with $k=\left(36\pi\right)^{1/3}$.

For steady state analysis of the granuloma model, it turns out to be convenient to sometimes use other differential equations, derived from those of model . The first is a differential equation for the volume of the lesion *V*, by using $V'=\rho\left(M_{u}'+M_{i}'\right)$:

$$V'=\rho\left(iA-dA\frac{M_{u}}{V}+bR_{MF}M_{i}-bM_{i}\right)$$

The second is a differential equation for the diameter of the lesion *L*, by using $V=\pi L^{3}/6$:

$L'=\frac{dL}{dV}\frac{dV}{dt}=\left(\frac{2}{\pi}\left(\frac{6}{\pi}V\right)^{-2/3}\right)\frac{dV}{dt}$ , so

$$L'=2\rho i-2d+\frac{12\rho dM_{i}}{\pi L^{3}}+\frac{2\rho b\left(R_{MF}-1\right)M_{i}}{\pi L^{2}}.$$

Because both *V* and *L* are determined by the sum of $M_{u}$ and $M_{i}$, any of the two equations can be replaced by Equation or without loss of information. In the main text, this has been done to make some of the figures.

Steady states

Starting with Equations and solving $M_{i}'=0$, we find the following steady state solutions

$$M_{i}^{*}=0\text{ or }M_{u}^{*}=A^{*}/T_{MAP}$$

Filling the first solution $M_{i}^{*}=0$ into the equation $M_{u}'=0$, using the relations $V=\rho\left(M_{u}+M_{i}\right)$ and $A=kV^{2/3}$, we obtain

$M_{u}'=\left(i-d\right)k\left(\rho M_{u}\right)^{2/3}/\rho$,

from which the steady state solution is $M_{u}^{*}=0$. Thus, the first steady state is the infection-free steady state:

- **steady state G1**: $\left(M_{u}^{*},M_{i}^{*}\right)_{\text{G1}}=\left(0,0\right)$

We now continue with the other steady-state solution of Equation , $M_{u}^{*}=A^{*}/T_{MAP}$. Filling this solution into Equation , using $M_{i}=V/\rho-M_{u}$, $V=\pi L^{3}/6$ and $A=\pi L^{2}$, we arrive at the following equation to be solved to obtain the steady state solution for *L*:

$$b\left(R_{MF}-1\right)T_{MAP}L^{2}+6\rho\left(iT_{MAP}-b\left(R_{MF}-1\right)\right)L-36\rho d=0$$

This equation has two solutions for *L*:

- **steady state G2**: $\left(M_{u}^{*},L^{*}\right)_{\text{G2}}=\left(\frac{A^{*}}{T_{MAP}},\frac{-\beta-\sqrt{\beta^{2}-4\alpha\gamma}}{2\alpha}\right)$
- **steady state G3**: $\left(M_{u}^{*},L^{*}\right)_{\text{G3}}=\left(\frac{A^{*}}{T_{MAP}},\frac{-\beta+\sqrt{\beta^{2}-4\alpha\gamma}}{2\alpha}\right)$ ,

in which

$$\begin{matrix} \alpha=bT_{MAP}\left(R_{MF}-1\right) \\ \beta=6\rho\left(iT_{MAP}-b\left(R_{MF}-1\right)\right) \\ \gamma=-36\rho d \end{matrix}$$

In the main text we refer to these steady states by their volume *V*, with *V_small_* as the volume of steady state G3 and *V_large_* as the volume of steady state G2.

*Conditions for existence of (a positive) steady state G2*

First, the volume occupied by the uninfected macrophages $\rho M_{u}^{*}$ should not exceed the volume of the total granuloma $V^{*}=\pi L^{*3}/6$ :

$$\begin{matrix} \rho\pi L^{*2}/T_{MAP}\leq\pi L^{*3}/6\Leftrightarrow\\ L^{*}\geq6\rho/T_{MAP}\Leftrightarrow\end{matrix}$$

$$\frac{-6\rho\left(iT_{MAP}-b\left(R_{MF}-1\right)\right)-6\sqrt{\rho^{2}\left(iT_{MAP}-b\left(R_{MF}-1\right)\right)^{2}+4\rho bdT_{MAP}\left(R_{MF}-1\right)}}{2bT_{MAP}\left(R_{MF}-1\right)}\geq\frac{6\rho}{T_{MAP}}.$$

If $R_{MF}>1$, condition never holds, as the left hand side is always negative.

If $R_{MF}<1$, condition holds if

$$b\left(1-R_{MF}\right)-iT_{MAP}\leq\sqrt{\left(iT_{MAP}+b\left(1-R_{MF}\right)\right)^{2}+4bdT_{MAP}\left(R_{MF}-1\right)/\rho}\text{,}$$

which holds if the left-hand side of is negative, i.e. if $i\geq b\left(1-R_{MF}\right)/T_{MAP}$, or if the left-hand side of is positive and

$$\begin{matrix} \left(b\left(1-R_{MF}\right)\right)^{2}-2b\left(1-R_{MF}\right)iT_{MAP}+\left(iT_{MAP}\right)^{2}\leq\\ \left(b\left(1-R_{MF}\right)\right)^{2}+2b\left(1-R_{MF}\right)iT_{MAP}+\left(iT_{MAP}\right)^{2}-4bd\left(1-R_{MF}\right)T_{MAP}/\rho\\ \Leftrightarrow-4b\left(1-R_{MF}\right)iT_{MAP}\leq-4bd\left(1-R_{MF}\right)T_{MAP}/\rho\\ \Leftrightarrow i\geq d/\rho\text{,} \end{matrix}$$

which does not hold because we defined $i<d/\rho$.

Summarising, the condition that the volume occupied by the uninfected macrophages should not exceed the total volume of the granuloma results in the following condition for existence of steady state G2:

$$\begin{matrix} R_{MF}<1\text{, and} \\ i\geq b\left(1-R_{MF}\right)/T_{MAP} \end{matrix}$$

Second, $L^{*}$ should be real:

$$\begin{matrix} \beta^{2}\geq4\alpha\gamma\\ \Leftrightarrow\left(6\rho\left(iT_{MAP}-b\left(R_{MF}-1\right)\right)\right)^{2}\geq-144\rho bdT_{MAP}\left(R_{MF}-1\right). \end{matrix}$$

If $R_{MF}>1$, that is always the case.

If $R_{MF}<1$,

$$\begin{matrix} \left(b\left(1-R_{MF}\right)\right)^{2}+2b\left(1-R_{MF}\right)iT_{MAP}+\left(iT_{MAP}\right)^{2}\geq4bd\left(1-R_{MF}\right)T_{MAP}/\rho\\ \Leftrightarrow\frac{d}{\rho}\leq\frac{b\left(1-R_{MF}\right)}{4T_{MAP}}+\frac{i}{2}+\frac{i^{2}T_{MAP}}{4b\left(1-R_{MF}\right)} \\ \Leftrightarrow\frac{d}{\rho}\leq i+\frac{T_{MAP}}{4b\left(1-R_{MF}\right)}\left(\left(\frac{b\left(1-R_{MF}\right)}{T_{MAP}}\right)^{2}-2i\frac{b\left(1-R_{MF}\right)}{T_{MAP}}+i^{2}\right) \\ \Leftrightarrow\frac{d}{\rho}\leq i+\frac{T_{MAP}}{4b\left(1-R_{MF}\right)}\left(\frac{b\left(1-R_{MF}\right)}{T_{MAP}}-i\right)^{2}. \end{matrix}$$

Taking all conditions together:

- **Conditions for existence of steady state G2 are**

$$\begin{matrix} R_{MF}<1\text{, and} \\ \frac{b\left(1-R_{MF}\right)}{T_{MAP}}\leq i<\frac{d}{\rho}\leq i+\frac{T_{MAP}}{4b\left(1-R_{MF}\right)}\left(\frac{b\left(1-R_{MF}\right)}{T_{MAP}}-i\right)^{2}. \end{matrix}$$

*Conditions for existence of (a positive) steady state G3*

First, the volume occupied by the uninfected macrophages $\rho M_{u}^{*}$ should not exceed the volume of the total granuloma $V^{*}=\pi L^{*3}/6$ :

$$\begin{matrix} \rho\pi L^{*2}/T_{MAP}\leq\pi L^{*3}/6\Leftrightarrow\\ L^{*}\geq6\rho/T_{MAP}\Leftrightarrow\end{matrix}$$

$$\frac{-6\rho\left(iT_{MAP}-b\left(R_{MF}-1\right)\right)+6\sqrt{\rho^{2}\left(iT_{MAP}-b\left(R_{MF}-1\right)\right)^{2}+4\rho bdT_{MAP}\left(R_{MF}-1\right)}}{2bT_{MAP}\left(R_{MF}-1\right)}\geq\frac{6\rho}{T_{MAP}}$$

If $R_{MF}>1$, condition holds if

$$\begin{matrix} b\left(R_{MF}-1\right)-iT_{MAP}+\sqrt{\left(iT_{MAP}-b\left(R_{MF}-1\right)\right)^{2}+4bdT_{MAP}\left(R_{MF}-1\right)/\rho}\geq2b\left(R_{MF}-1\right) \\ \Leftrightarrow\sqrt{\left(iT_{MAP}-b\left(R_{MF}-1\right)\right)^{2}+4bdT_{MAP}\left(R_{MF}-1\right)/\rho}\geq iT_{MAP}+b\left(R_{MF}-1\right) \\ \Leftrightarrow\left(iT_{MAP}\right)^{2}+\left(b\left(R_{MF}-1\right)\right)^{2}-2biT_{MAP}\left(R_{MF}-1\right)+4bdT_{MAP}\left(R_{MF}-1\right)/\rho\geq\\ \left(iT_{MAP}\right)^{2}+\left(b\left(R_{MF}-1\right)\right)^{2}+2biT_{MAP}\left(R_{MF}-1\right) \\ \Leftrightarrow4bdT_{MAP}\left(R_{MF}-1\right)/\rho\geq4biT_{MAP}\left(R_{MF}-1\right) \\ \Leftrightarrow d/\rho\geq i\text{,} \end{matrix}$$

which is always the case.

If $R_{MF}<1$, condition holds if

$$iT_{MAP}-b\left(1-R_{MF}\right)\geq\sqrt{\left(iT_{MAP}+b\left(1-R_{MF}\right)\right)^{2}-4bdT_{MAP}\left(1-R_{MF}\right)/\rho}\text{,}$$

which holds if the left-hand side is positive, i.e. if $iT_{MAP}\geq b\left(1-R_{MF}\right)$, and if:

$$\begin{matrix} \left(b\left(1-R_{MF}\right)\right)^{2}-2b\left(1-R_{MF}\right)iT_{MAP}+\left(iT_{MAP}\right)^{2}\geq\\ \left(b\left(1-R_{MF}\right)\right)^{2}+2b\left(1-R_{MF}\right)iT_{MAP}+\left(iT_{MAP}\right)^{2}-4bd\left(1-R_{MF}\right)T_{MAP}/\rho\\ \Leftrightarrow-4b\left(1-R_{MF}\right)iT_{MAP}\geq-4bd\left(1-R_{MF}\right)T_{MAP}/\rho\\ \Leftrightarrow i\leq d/\rho\text{,} \end{matrix}$$

which always holds because we defined $i<d/\rho$.

Summarising, the condition that the volume occupied by the uninfected macrophages should not exceed the total volume of the granuloma results in the following condition for existence of steady state G3:

$$R_{MF}>1\text{,}$$

or

$$\begin{matrix} R_{MF}<1\text{, and} \\ i\geq b\left(1-R_{MF}\right)/T_{MAP}. \end{matrix}$$

Second, $L^{*}$ should be real: see conditions for steady state G2.

Taking all conditions together:

- **Conditions for existence of steady state G3 are**

$$\begin{matrix} R_{MF}>1\text{, or} \\ \\ R_{MF}<1\text{, and} \\ \frac{b\left(1-R_{MF}\right)}{T_{MAP}}\leq i<\frac{d}{\rho}\leq i+\frac{T_{MAP}}{4b\left(1-R_{MF}\right)}\left(\frac{b\left(1-R_{MF}\right)}{T_{MAP}}-i\right)^{2} \end{matrix}$$

Local stability of the steady states

For stability analysis, we use the system expressed in differential equations for *L* (Equation ) and *M_i_*, reformulated from Equation :

$$M_{i}'=b\frac{T_{MAP}}{\pi L^{2}}M_{i}\left(\frac{\pi L^{3}}{6\rho}-M_{i}\right)-bM_{i}$$

$$L'=2\rho i-2d+\frac{12\rho dM_{i}}{\pi L^{3}}+\frac{2\rho b\left(R_{MF}-1\right)M_{i}}{\pi L^{2}}$$

Local stability analysis is done by determining the Jacobian matrices in the three steady states, and calculating the traces and determinants of these matrices. Steady states are stable if the trace is negative and the determinant is positive. The general Jacobian matrix reads

*Local stability of steady state G1*

In the system of Equations and , the infection-free steady state G1 is *not* a steady state mathematically, i.e. it is not a solution to the equations $M_{i}'=0$ and $L'=0$. That is because of the terms $2\rho i-2d$ in the equation for *L*. However, we have assumed in our model that $i<d/\rho$ to prevent growth of a granuloma in absence of infected macrophages. Hence, in any case,

$$L'\leq\frac{12dM_{i}}{\pi L^{3}}+\frac{2b\left(R_{MF}-1\right)M_{i}}{\pi L^{2}}\text{,}$$

which means that if the infection-free steady state is stable with $i=d/\rho$, it will also be stable for $i<d/\rho$. If $i=d/\rho$, the system of equations above does have G1 as the infection-free steady state, so the Jacobian matrix can be used to study local stability for this special case.

The Jacobian matrix is rewritten by using $V=\pi L^{3}/6$, and defining $m_{i}=M_{i}/\left(M_{u}+M_{i}\right)=\rho M_{i}/V$:

$$J=\left(\begin{matrix} -b\left(1-\frac{LT_{MAP}}{6\rho}\left(1-2m_{i}\right)\right) & \frac{bm_{i}VT_{MAP}}{6\rho^{2}}\left(1+2m_{i}\right) \\ \frac{\rho}{V}\left(2d+\frac{bL\left(R_{MF}-1\right)}{3}\right) & -2m_{i}\left(\frac{3d}{L}+\frac{b\left(R_{MF}-1\right)}{3}\right) \end{matrix}\right)$$

Local stability requires the Trace to be negative and the Determinant to be non-negative:

$$\text{Tr}_{\text{G1}}=-b\left(1-\frac{LT_{map}}{6\rho}\left(1-2m_{i}\right)\right)-2m_{i}\left(\frac{3d}{L}+\frac{b\left(R_{MF}-1\right)}{3}\right)$$

By definition, $0\leq m_{i}\leq1$. If $m_{i}=0$ (and $L=0$), then $\text{Tr}_{\text{G1}}$ reduces to $-b-6dm_{i}/L$, which is always negative. If $m_{i}>0$, then the term $-6dm_{i}/L\to-\infty$. Thus, $\text{Tr}_{\text{G1}}$ is always negative.

$$\begin{matrix} \text{Det}_{\text{G1}}=2bm_{i}\left(1-\frac{LT_{MAP}\left(1-2m_{i}\right)}{6\rho}\right)\left(\frac{3d}{L}+\frac{b\left(R_{MF}-1\right)}{3}\right) \\ -\frac{bm_{i}T_{MAP}}{6\rho}\left(2d+\frac{bL\left(R_{MF}-1\right)}{3}\right)\left(1+2m_{i}\right) \end{matrix}$$

By definition, $0\leq m_{i}\leq1$. If $m_{i}=0$, then Det_G1_ reduces to $6bdm_{i}/L$, which is always positive. If $m_{i}>0$, then the term $6bdm_{i}/L\to+\infty$. Thus, the determinant is always positive.

**Conclusion about steady state G1**: the infection-free steady state G1 always exists, and is always locally stable

*Local stability of steady state G2*

For steady state G2, the Jacobian matrix is rewritten by using $M_{u}^{*}=A^{*}/T_{MAP}$, $A=\pi L^{2}$, $V=\pi L^{3}/6$, and defining $m_{u}=M_{u}/\left(M_{u}+M_{i}\right)=1-m_{i}$:

$$J_{\text{G2}}=\left(\begin{matrix} -\frac{b}{m_{u}^{*}}\left(1-m_{u}^{*}\right) & \frac{bA^{*}}{6\rho m_{u}^{*}}\left(1-m_{u}^{*}\right)\left(3-2m_{u}^{*}\right) \\ 2\rho\left(\frac{d}{V^{*}}+\frac{b\left(R_{MF}-1\right)}{A^{*}}\right) & -\left(1-m_{u}^{*}\right)\left(\frac{dA^{*}}{V^{*}}+\frac{2b\left(R_{MF}-1\right)}{3}\right) \end{matrix}\right)$$

Local stability requires $\text{Tr}_{\text{G2}}$ to be negative and $\text{Det}_{\text{G2}}$ to be non-negative:

In steady state G2, $0<m_{u}^{*}<1$, so $\text{Tr}_{\text{G2}}$ is always negative.

The determinant of G2 is

$\text{Det}_{\text{G2}}\text{ }=\frac{-b\left(1-m_{u}^{*}\right)}{3m_{u}^{*}}\left(\frac{m_{u}^{*}dA^{*}}{V^{*}}+b\left(R_{MF}-1\right)\right)$.

The steady state only exists if $R_{MF}<1$, in which case $\text{Det}_{\text{G2}}$ is positive if

$$\begin{matrix} \frac{m_{u}^{*}dA^{*}}{V^{*}}+b\left(R_{MF}-1\right)<0 \\ \Leftrightarrow b\left(1-R_{MF}\right)>\frac{36\rho d}{L^{*2}T_{MAP}} \\ \Leftrightarrow\text{ }L^{*2}>\frac{36d}{bT_{map}\left(1-R_{mf}\right)}=\frac{\gamma}{\alpha} \end{matrix}$$

with *γ* and *α* as defined above for steady states G2 and G3. Because in steady state G2, $L^{*}\geq-\beta/2\alpha$, so $L^{*2}\geq\left(\beta/2\alpha\right)^{2}$, and because for the steady state to exist, $\beta^{2}-4\alpha\gamma>0$, which is equivalent to $\left(\beta/2\alpha\right)^{2}>\gamma/\alpha$, it can be concluded that $L^{*2}>\gamma/\alpha$. Thus, $\text{Det}_{\text{G2}}$ is always positive.

**Conclusion about steady state G2**: the infected steady state G2 exists if

$$\begin{matrix} R_{MF}<1\text{, and} \\ \frac{b\left(1-R_{MF}\right)}{T_{MAP}}\leq i<\frac{d}{\rho}\leq i+\frac{T_{MAP}}{4b\left(1-R_{MF}\right)}\left(\frac{b\left(1-R_{MF}\right)}{T_{MAP}}-i\right)^{2}, \end{matrix}$$

in which case it is always locally stable.

*Local stability of steady state G3*

For steady state G3, the Jacobian matrix is rewritten as for G2:

$$J_{\text{G3}}=\left(\begin{matrix} -\frac{b}{m_{u}^{*}}\left(1-m_{u}^{*}\right) & \frac{bA^{*}}{6\rho m_{u}^{*}}\left(1-m_{u}^{*}\right)\left(3-2m_{u}^{*}\right) \\ 2\rho\left(\frac{d}{V^{*}}+\frac{b\left(R_{MF}-1\right)}{A^{*}}\right) & -\left(1-m_{u}^{*}\right)\left(\frac{dA^{*}}{V^{*}}+\frac{2b\left(R_{MF}-1\right)}{3}\right) \end{matrix}\right)$$

Local stability requires $\text{Tr}_{\text{G3}}$ to be negative and $\text{Det}_{\text{G3}}$ to be non-negative:

In steady state G3, $0<m_{u}^{*}<1$, so $\text{Tr}_{\text{G3}}$ is always negative.

The determinant of G3 is

$\text{Det}_{\text{G3}}\text{ }=\frac{-b\left(1-m_{u}^{*}\right)}{3m_{u}^{*}}\left(\frac{m_{u}^{*}dA^{*}}{V^{*}}+b\left(R_{MF}-1\right)\right)$.

If $R_{MF}>1$, then $\text{Det}_{\text{G3}}$ is always negative, so steady state G3 is unstable. If $R_{MF}<1$, then $\text{Det}_{\text{G3}}$ is positive if

$$\begin{matrix} \frac{m_{u}^{*}dA^{*}}{V^{*}}+b\left(R_{MF}-1\right)<0 \\ \Leftrightarrow b\left(1-R_{MF}\right)>\frac{36\rho d}{L^{*2}T_{MAP}} \\ \Leftrightarrow\text{ }L^{*2}>\frac{36d}{bT_{map}\left(1-R_{mf}\right)} \\ \Leftrightarrow L^{*}>\sqrt{\frac{\gamma}{\alpha}} \\ \Leftrightarrow-\frac{\beta}{2\alpha}-\sqrt{\left(\frac{\beta}{2\alpha}\right)^{2}-\frac{\gamma}{\alpha}}>\sqrt{\frac{\gamma}{\alpha}} \\ \Leftrightarrow-\frac{\beta}{2\alpha}-\sqrt{\frac{\gamma}{\alpha}}>\sqrt{\left(\frac{\beta}{2\alpha}\right)^{2}-\frac{\gamma}{\alpha}} \\ \Leftrightarrow\left(\frac{\beta}{2\alpha}\right)^{2}+\frac{\beta}{\alpha}\sqrt{\frac{\gamma}{\alpha}}+\frac{\gamma}{\alpha}>\left(\frac{\beta}{2\alpha}\right)^{2}-\frac{\gamma}{\alpha} \\ \Leftrightarrow\frac{\beta^{2}\gamma}{\alpha^{3}}<\frac{4\gamma^{2}}{\alpha^{2}} \\ \Leftrightarrow\beta^{2}-4\alpha\gamma<0, \end{matrix}$$

which is never the case, because for the steady state to exist, $\beta^{2}-4\alpha\gamma$ should be positive. Therefore, also if $R_{MF}<1$, $\text{Det}_{\text{G3}}$ is negative.

**Conclusion about steady state G3**: the infected steady state G2 exists if

$$\begin{matrix} R_{MF}>1\text{, or} \\ \\ R_{MF}<1\text{, and} \\ \frac{b\left(1-R_{MF}\right)}{T_{MAP}}\leq i<\frac{d}{\rho}\leq i+\frac{T_{MAP}}{4b\left(1-R_{MF}\right)}\left(\frac{b\left(1-R_{MF}\right)}{T_{MAP}}-i\right)^{2}\text{,} \end{matrix}$$

in which case it is never stable.
